# Silvopastoral systems for offsetting livestock emissions in the tropics: a case study of a dairy farm in Costa Rica

**DOI:** 10.1007/s13593-022-00834-z

**Published:** 2022-10-13

**Authors:** Robert Brook, Eilidh Forster, David Styles, André Mancebo Mazzetto, Claudia Arndt, M. Jimena Esquivel, David Chadwick

**Affiliations:** 1 School of Natural Sciences, Bangor University, Bangor, LL57 2UW UK; 2 CATIE-Centro Agronómico Tropical de Investigación y Enseñanza, Turrialba, Costa Rica; 3 School of Engineering, University of Limerick, Limerick, Ireland; 4 Ryan Institute, School of Biological & Chemical Sciences, University of Galway, Galway, Ireland; 5 AgResearch Limited, Lincoln, New Zealand; 6 Mazangira Centre, International Livestock Research Institute (ILRI), P.O. Box 30709-00100, Nairobi, Kenya; 7 Institute of Environmental Sciences, Faculty of Sciences, Leiden University, Leiden, The Netherlands; 8 Department of Animal Sciences, Wageningen University, Wageningen, The Netherlands

**Keywords:** Life cycle assessment, Carbon sequestration, Milk, Trees, Live fences, Agroforestry

## Abstract

Ways are being sought to reduce the environmental impact of ruminant livestock farming. Integration of trees into farming systems has been advocated as a measure to deliver ecosystem services, inter alia climate regulation and adaptation, water quality regulation, provisioning of fibre, fuel and habitats to support biodiversity. Despite the rapid expansion of cattle farming in the tropics, notably in Latin America, there is little robust evidence on the extent to which trees are able to mitigate the effects of cattle farming in this ecological zone. This article describes a case study conducted on a large, specialised dairy farm in Costa Rica, where two-thirds of the field boundaries are live tree fences. For the first time, this study quantifies the offset potential of trees by estimating rate of carbon sequestration in a silvopastoral system (SPS) in the tropics. It was found that over a 30-month interval, trees sequestered 1.43 Mg C ha^−1^ year^−1^ above and below ground. Attributional life cycle assessment (LCA) (cradle to farm gate) was applied to calculate the carbon footprint of milk produced on the farm for the years 2016 to 2018. Trees in live fences offset 21–37% of milk footprints, resulting in residual net footprints of 0.75±0.25 to 0.84±0.26 kg CO_2_ eq. kg^−1^ milk. Exclusion of life cycle emissions that may not fall within national emission inventory accounting (e.g. fertiliser manufacture and feed production) increased the mean offset from 27 to 34% of gross milk footprint. Although based on temporally limited data (30 months), our findings indicate that a live fence SPS could play an important role in short- to medium-term climate mitigation from livestock production, buying time for deployment of long-term mitigation and adaptation planning.

**Supplementary Information:**

The online version contains supplementary material available at 10.1007/s13593-022-00834-z.

## Introduction

Recent discussions about the environmental, climatic and social effects of agriculture and various high-level reports have argued that ‘Food systems are at a crossroads. A profound transformation is needed at all scales…’ (HLPE 2019) and that ‘…planetary boundaries are reached and exceeded…’ (Sinclair et al. 2019). Livestock systems, especially ruminants, are attracting increasing scrutiny. The world’s livestock sector, directly and indirectly, utilises 83% of the world’s cultivated and grazed land and contributes 56–58% of food’s various greenhouse gas (GHG) emissions (Poore and Nemecek 2018). Other concerns include eutrophication, acidification and widespread land use change (Van Zanten et al. 2019). These issues have spawned a range of perspectives about future scenarios for livestock farming (Röös et al. 2017) and the role of livestock in circular food systems (Van Zanten et al. 2019).

As part of the response to concerns about adverse environmental effects of agriculture, silvopastoral systems (SPS) are being advocated in many quarters (Broom 2017; Chará et al. 2019). Incorporation of trees into pastoral systems is reported to have numerous benefits including sequestration of atmospheric CO_2_, provision of shade (in hot climates), browse, fodder, wood and fibre, shelter from wind, stabilisation of soil, biological nitrogen fixation when trees are hosts for N_2_-fixing symbionts, and acquisition of nutrients from the soil which are available to accompanying crops via leaf and fine root senescence. Typically in Central America, SPS trees are grown as live fences around field boundaries, and often remnant trees are left in pastures to provide browse and shade (Montagnini et al. 2013). It is reported that the inclusion of trees in pastoral systems is compatible with maintaining milk outputs in the tropics (Paciullo et al. 2014)

Many of the papers on SPS in the tropics report carbon (C) stocks in soil and woody biomass. Hoosbeek et al. (2018) and Cárdenas et al. (2019) are examples from Central America, but none has looked at rates of C sequestration by trees over time; thus, they are snapshots from which it is impossible to deduce to what extent trees compensate for GHG emissions. Furthermore, in tropical systems, there are no articles in the literature which have combined an analysis of environmental costs of dairy production and rates of net C fixation by trees over time to arrive at an estimate of mitigation potential for environmental footprints as calculated using life cycle assessment (LCA).

Life cycle assessment is an internationally accepted method for assessing the environmental impact of products and services delivered (ISO 14040 2006), based on a systems approach that assesses the impact of a product throughout its entire life cycle. Depending on the objectives of the analysis and the way it is implemented, LCA can enable identification of emissions ‘hotspots’ within a process, compare between the environmental impact of different systems or management regimes and calculate the environmental footprint of livestock products, such as milk (Styles et al. 2018; Thomassen et al. 2008). Life cycle assessment is an increasingly used decision-support tool for policymakers, industry and the public (Notarnicola et al. 2017). It considers upstream and downstream effects of production and end-of-life, in addition to product use impacts, thus enabling improvement strategies to be effectively targeted within supply chains and providing a transparent evidence base to inform responsible consumption and/or procurement decisions (Notarnicola et al. 2017).

There are a small number of studies published which included SPS as a management option when calculating the C footprint of milk production based on livestock emissions, but not C sequestration. Esteban Rivera et al. (2016) conducted a comparative LCA of an intensive dairy SPS with a high density of *Leucaena leucocephala* (Lam.) de Wit shrubs (> 8000 ha^−1^) and a conventional pastoral system without trees, in Colombia. They found that to produce 1 kg of fat- and protein-corrected milk (FPCM), the intensive SPS emitted less carbon dioxide equivalent (CO_2_ eq) than the conventional system without trees (2.05 vs 2.34 kg CO_2_ eq, respectively). Using the IPCC Tier 2 methodology, Parra and Mora-Delgado (2019) in Colombia found that the methane (CH_4_) emission intensity (g CH_4_ kg^−1^ milk) from dairy Holstein cattle was greater in intensive pastures than in a live fence SPS (11.0 and 8.4 g CH_4_ L^−1^ milk, respectively). In contrast, Molina-Rivera et al. (2019) in Mexico found no differences in greenhouse gas emissions determined by LCA between cattle grazing in monoculture, montane and intensive SPS (with 5,000 ha^−1^ *Leucaena leucocephala* shrubs) systems. However, none of these studies accounted for the greenhouse gas offset potential of C sequestration of growing trees in the SPS.

This article examines the environmental costs of milk production from a commercial dairy farm in Costa Rica, and in particular the extent to which the C footprint of milk production can be offset by C sequestration in live fences grown on the farm. A unique aspect of this LCA study is the integration of measurements of C sequestration in live fences in a tropical dairy farming system over a 2.5-year interval. The implications of the findings for development of low environmental impact dairy systems in the tropics are discussed.

## Materials and methods

### Case study farm

**Fig. 1:**
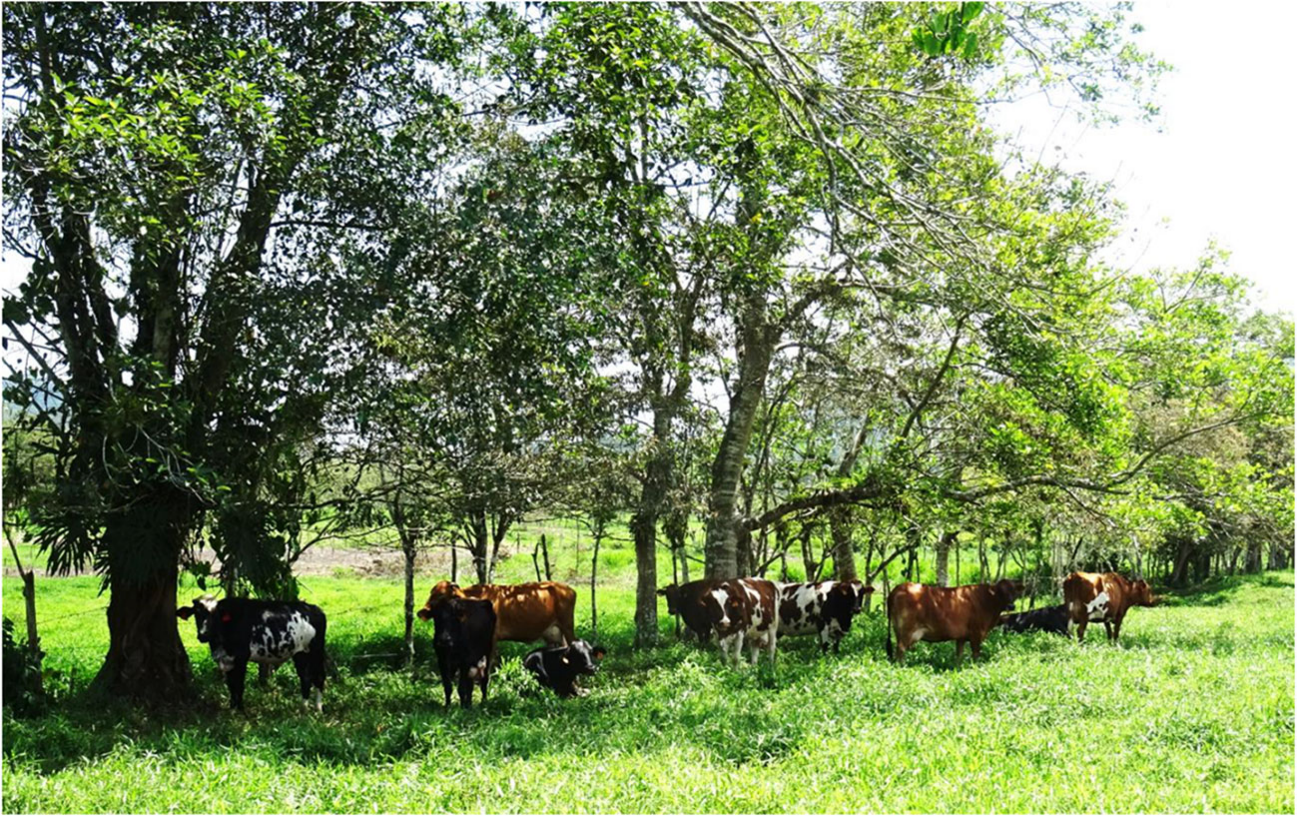
Cattle under live fence shade, CATIE dairy farm, Costa Rica.

The research took place on the commercial dairy farm of the Tropical Agricultural Research and Higher Education Center (CATIE, Spanish acronym) in Costa Rica (Fig. 1). CATIE is located near Turrialba, Cartago Province (latitude 9°54′N, longitude 83°41′W) and the 62.1-ha dairy farm forms part of a larger enterprise comprising multiple operations, including dairy, beef and cash crops (predominantly sugar cane and coffee). The farm is 630 m above sea level and experiences a tropical monsoon climate ameliorated by its mid-altitude location (Köppen classification Am). Average temperature is 22.6°C, and annual precipitation is 2600 mm with a short drier period in February and March. In terms of the dairy farm classification utilised in Mazzetto et al. (2020) and Duffy et al. (2021), the farm is intermediate between lowland and upland specialised dairy, but is much larger than the average for Costa Rica in both area and herd size. Cattle are pasture fed (with supplements during milking) and are not housed, and therefore, it is not classified as being highly intensive.

**Fig. 2:**
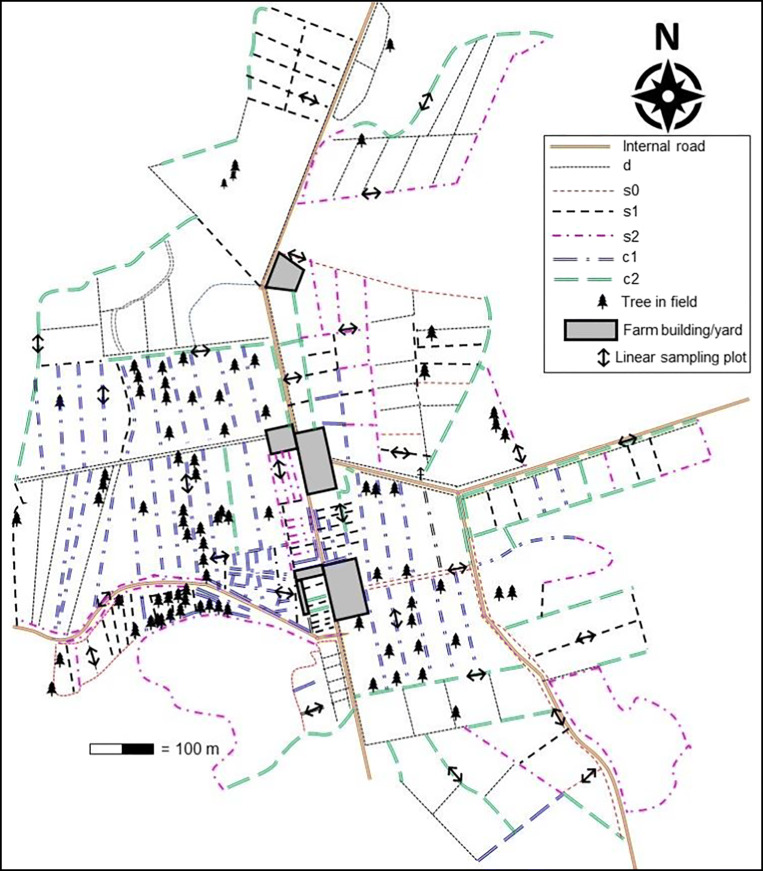
Dairy farm map showing fence types, mature trees in fields and locations of sample plots. d = wire fence with dead posts; s0 = simple live fence, no canopy; s1 = simple live fence, single species; s2 = simple live fence, mixed species; c1 = complex live fence, single species; c2 = complex live fence, mixed species.

It is important to emphasise that this case study was not a designed experiment, and the recordings were taken from operations on a working farm. The farm manager took all management decisions for the farm; thus, the findings are representative of a large commercial farm at mid-altitude Costa Rica. The dairy farm is divided into 165 paddocks with a wide range of areas (Fig. 2) divided by a combination of post and wire fences (using purchased preserved wooden posts) and live fences. Live fences are where living tree stems are used as supports for wire, and on this farm, they vary greatly in age and structure (Table 1). Most of the trees in live fences were established by vegetative reproduction, cutting branches from older trees (2.0–2.5 m long and 5–10 cm diameter), storing the stems in shade for at least 3 weeks to promote potential rooting and planting them directly into the pasture, 30–40 cm deep, along the fence line at about 2.0 m apart. A second in-filling planting of 15% of new cuttings is generally expected. Partial or total pruning at about 2.0 m height was done after approximately 2 years to encourage branching.

**Table 1:** Quantity and description of each fence type within the dairy system in 2016 and number of surviving trees per 10 m length in 2019.

Fence type	Total length (m)	% of total fences	Description of fence	Number of live trees per 10 m in 2019	Survival between Sept 2016 and Mar 2019 (%)
d	10,643	32.2%	Dead (preserved posts and wire)	None	
s0	2,193	6.6%	Simple live fence, no canopy (recently pollarded or planted with little or no sign of regrowth)	0.8	23
s1	3,813	11.5%	Simple live fence, single species (height <6m and canopy diameter <4m)	1.5	75
s2	5,414	16.4%	Simple live fence, mixed species (height <6m and canopy diameter <4m)	1.8	56
c1	5,854	17.7%	Complex live fence (i.e. multi-strata), mono species (height >6m and canopy diameter >4m)	2.4	65
c2	5,165	15.6%	Complex live fence (i.e. multi-strata), multi species (height >6m and canopy diameter >4m)	2.7	73

Cows are predominantly dairy breeds (Jersey, Holstein), dual-purpose (e.g. Simental), crossed with more heat resistant and zebu breeds (e.g., Brahman, Gyr), with an average of 133 lactating cows over the study period (Table 2). The main pasture grass species are tanner grass (*Brachiaria arrecta* (Hack. ex T. Durand & Schinz) Sten. syn. *Urochloa arrecta* (Hack. ex T. Durand & Schinz) Morrone & Zuloaga) and star grass (*Cynodon nlemfuensis* Vanderyst), both being C4 species. The herd structure is typical for Costa Rica and exhibits no unusual features (Mazzetto et al. 2020). The working herd averaged 324 animals during the period studied and is managed in four different cohorts according to their differing needs. Over half of these were in production or between lactation cycles (dry cows); and the remainder were made up of calves, heifers and three bulls. Male calves produced as a co-product of the dairy system are not considered part of the working herd. They are sold at auction at around 30 days of age. All excreta from the collection yard and milking parlour is collected and mechanically separated into solid and liquid fractions. The liquid fraction is anaerobically digested and the methane is used to generate electricity to supplement the mains supply. The digestate is applied to the pastures closest to the dairy and the solids are used on other crops on the farm. Although pasture grows all year round, there are seasonal variations and lactating cows receive supplements (mostly maize and soya concentrates plus cut sugar cane) generally constituting about 20% of dry matter intake, particularly when pasture is insufficient (Table 2).

**Table 2:** Inventory of major inputs and outputs each year and GHG emissions for each year with biomass carbon offset, expressed as CO_2_ equivalents from the silvopastoral farm system. Offsets calculated as a percentage of dairy carbon footprint (LCA approach) and as a percentage of national inventoried emissions (i.e. excluding production of feeds and fertilizers usually imported to Costa Rica). *Applied to 33 ha of improved pasture on the farm. ^#^From applications and deposition of N fertilisers, digestate, solid fraction of separated excreta, urine and dung deposited by grazing cattle.

Parameter	Unit	2016	2017	2018	Source
Number lactating cows	head	141	124	135	Farm records
Farm milk production	kg FPCM	935,752	810,237	900,664	Farm records
Milk per cow	kg FPCM	6637	6534	6672	Farm records
Average lactation period	days	310	320	312	Farm records
Non-productive cattle on farm	head	168	204	201	Farm records
Calves sold	head	95	77	95	Farm records
Cows sold	head	47	47	47	Farm records
Concentrate feed	kg	410,320	286,074	375,820	Farm records
Forage dry matter intake	kg	1,210,632	1,328,668	1,314,829	Tier 2 estimated gross energy intake (IPCC 2006) minus energy in concentrate feed
Electricity	kWh	58,777	56,959	62,012	Farm records
Diesel	L	5,142	4,522	4,924	Farm records
Petrol (gasoline)	L	866	762	829	Farm records
Water	m^3^	7,000	7,300	7,300	Farm records
Urea-N app. rate*	kg/ha	30	40	59	Farm records
Ammonium-nitrate N app. rate*	kg/ha	86	117	167	Farm records
Fertiliser-P app. rate*	kg/ha	18	17	22	Farm records
Fertiliser-K app rate*	kg/ha	17	19	34	Farm records
Enteric methane	kg	31,059	31,691	32,565	Latin American Y_m_ factors (Mazzetto et al. 2020)
Manure management CH_4_	kg	696	767	752	Tier 2, IPCC (2006) based on volatile solids
Manure management N_2_O	kg	21	22	22	Tier 2, IPCC (2006) based on N excretion
Manure management NH_3_ (housing and storage)	kg	1,756	1,928	1,890	Misselbrook et al. (2015), based on N excretion
Soil nitrous oxide^#^	kg	219	250	241	Tier 1, IPCC (2019) based on N excretion plus applications
Soil NH_3_^#^	kg	1,418	1,567	1,531	Misselbrook et al. (2015) based on N applications
Soil NO_3_ leaching	kg	9,388	11,072	11,749	Duffy et al. (2018) 0.1 x N excreted and applied
Soil P leaching	kg	205	231	228	Styles et al. (2015) 0.03 x P excreted and applied
Farm level system GHG emissions (kg CO_2_ eq)					
Bought animals		0	0	0	
Enteric fermentation		776,469	792,274	814,132	
Manure management		23,595	25,782	25,395	
Soil N_2_O		100,096	121,239	139,331	
Imported feed		236,755	165,065	216,848	
Agrochemicals and energy		42,950	47,954	62,729	
LCA sub-total		1,179,864	1,152,314	1,258,434	
Carbon sequestration/offset					
Soil CO_2_		0	0	0	
Wood biomass CO_2_		−325,298	−325,298	−325,298	
LCA net total		854,566	827,016	933,136	
Offset		−28%	−28%	−26%	
Inventory total		919,320	956,200	997,263	
Inventory offset		−35%	−34%	−33%	

### Live fence carbon stock measurements

In September 2016, a complete survey of the paddock boundaries was conducted to characterise them as a prerequisite for development of a sampling strategy. There were a total of 33.1 km fence lines, of which 22.4 km was live fences. These were classified as described in Table 1 and located as presented in Fig. 2.

A sample plot was defined as a representative 20-m length of live fence, in which all trees were measured. Sample linear plots were identified on the map (Fig. 2) according to the following rules:

- Fence type d was not sampled as the purpose was to investigate the effects of live fences;
- No two plots were within 100 m of each other (to ensure independence of experimental units);
- Samples represented the wide range of tree numbers within each live fence.

Sample plots included at least five of each fence type of live fences (s0, s1, s2, c1 and c2). Particular emphasis was given to ensure that the live fences bordering paddocks grazed by lactating cows were fully represented during sampling. As the LCA also takes into account the environmental burden arising from non-productive cattle, i.e. dry cows and young stock, live fences used for these purposes at the time of the initial survey in 2016 were also sampled. The farm operates a rotational grazing system, so paddocks are allocated to particular purposes as appropriate for optimal management of the farm, and this varies over time.

There were 26 sample plots from which data were collected (Fig. 2). The number of trees per 20-m linear plot varied from three to 20. The species in the live fences (with proportion in parentheses) were as follows: *Erythrina fusca* Lour. (50.8%) and *E. poeppigiana* (Walp). O. F. Cook (6.4%) (Fabaceae), *Tricanthera gigantea* Nees. (Acanthaceae) (12.3%), *Gliricidia sepium* (Jacq.) Kunth exWalp. (Fabaceae) (20.3%), not identified (mostly woody cuttings without foliage) (10.1%).

The first sampling period was September and October 2016, when tree diameter at breast height (DBH: 1.40 m above the ground) and crown diameter in two directions were recorded. Crown diameter data were used to determine the area of land shaded by tree canopies in 2016. Diameter at breast height on the same trees was re-measured in March and April 2019, and for the purposes of the calculations presented here, recordings were considered to be 30 months apart. Trees growing in pastures (indicated in Fig. 2) but not in fence lines were not included in this study due to time constraints and because they were a minor component of standing biomass. There were 119 free standing trees, out of an estimated total population of 5430 trees in live fences in 2016.

In the absence of definitive farm records, time of live fence planting was estimated from Google Earth © images and by the stage of growth in September 2016 (Supplementary Information). Out of the 26 sampled linear plots, only four were visible before 2010. Between 2013 and 2016, live fences within our sample were established at a rate of about four per year. The majority of live fences were 6 years old or younger by the time of first assessment in 2016, and thus, most trees were in the phase of rapid biomass accumulation, apart from those which died.

Above and below ground biomass of trees was calculated based on DBH after Kuyah et al. (2012a, 2012b). Carbon content of oven dry wood was assumed to be 0.48 g C/g dry matter, a widely used conversion factor. Consultation of the Global Wood Density Database (Zanne et al. 2009) revealed that there was a twofold difference in wood density in the species growing in the live fences on the CATIE farm. Oven dry tree wood density for *Erythrina* spp. was 0.31, *Trichanthera gigantea* was 0.45 and *Gliricidia sepium* was 0.62 g cm^−3^. The algorithms of Kuyah et al. (2012a, 2012b) which incorporated wood density were used which improved the fit compared to using DBH alone

- Above ground biomass (kg) = 0.225*(DBH^2.341^)*(wood density^0.73^) (Eq. 1; r^2^ = 0.98);
- below ground biomass (kg) = 0.087*(DBH^2.257^)*(wood density^0.611^) (Eq. 2; r^2^ = 0.96);

where DBH is the diameter at breast height (cm) and the units of wood density are g cm^−3^.

Tree stems which had no canopy in 2016 (either had not yet sprouted or had been recently pollarded) and those which had died by September 2019 were classified as stumps, and height and diameter were recorded. For the calculation of biomass, they were assumed to be cylinders with a wood density of 0.52 g cm^−3^, the value used by Kuyah et al. (2012a) when wood density is not known. Calculations are presented in Supplementary Information.

### Life cycle assessment goal and scope

**Fig. 3:**
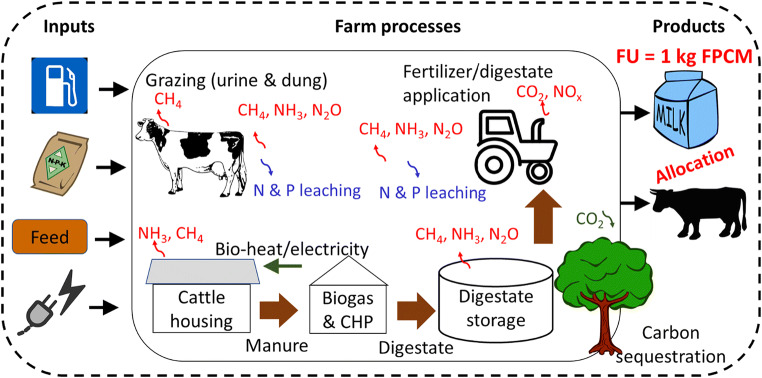
System boundary indicating the processes included in the LCA undertaken in this study to evaluate the global warming potential of the CATIE dairy farm, and the carbon footprint of the functional unit (FU)—1 kg of energy-corrected milk (ECM). Biogas produced from the manure in an anaerobic digestor generates bio-electricity and hot water for the dairy is produced from solar panels.

Attributional LCA (ISO 14040 2006) was undertaken to calculate total GHG emissions from the farm system (Fig. 3) and relate them to a functional unit of 1 kg FPCM, as per recent milk footprint studies (O’Brien et al. 2012; Mazzetto et al. 2020). System burdens (and tree growth carbon credits) were allocated across milk and co-products (sold calves and culled adult cows) according to gross energy contents of 2.5 and 12.4 MJ kg^−1^ FPCM and animal live weight, respectively (Soteriades et al. 2018). The boundaries of analysis are cradle to farm gate (includes upstream processes, production and transport of necessary inputs such as fertiliser and feed) (Fig. 3), as per Soteriades et al. (2018). Emissions associated with infrastructure are not accounted for. The temporal coverage of the LCA is a period of 3 years, 2016–2018. Only one environmental impact category was considered in this study owing to the focus on GHG emissions—global warming potential (GWP), expressed as CO_2_ eq. Specifically, carbon dioxide (CO_2_), methane (CH_4_) and nitrous oxide (N_2_O) are attributed GWP_100_ factors of 1, 25 and 298, respectively, in line with UNFCCC reporting guidelines for national GHG inventories (UNFCCC 2016).

We recognise that GWP_100_ is based on a time horizon of 100 years, whilst our biomass C sequestration offset potential is based on only 2.5 years of measured sequestration. Implications around this are discussed later. However, we apply the IPCC default land use change methodology recommended for national inventory reporting (IPCC 2006) and product carbon footprints (BSI 2011), assuming that the aforementioned C sequestration measured across trees of different ages, mostly established within the preceding 6 to 8 years, is representative of growth within the 20-year default period used to account for land use change. To do this, we extrapolated annual mean C sequestration of 1.43 Mg C ha^−1^ year^−1^ (cell Q244 on spreadsheet in Supplementary Information) to the 62.1 ha of farm with live fences, and included this as an average annual ‘land use change’ input at the farm system level (described further in the next section).

### Inventory data

Most of the activity data collected for the farm, such as quantity and type of fertiliser and feed imported and milk produced were provided by the farm manager from electronic farm records and from his personal knowledge of farm management practices (Table 2). Additional qualitative information was captured in the worksheet during interviews with the farm manager, for example all fertiliser and anaerobic digestate was surface broadcast. Manufacturer datasheets provided data on feed concentrate and fertiliser composition; and CATIE’s administration department provided data on farm (main grid) electricity usage. It was assumed that soil carbon is in equilibrium, and therefore was not measured given that pastures are long-term and land conversion occurred many decades ago. The Interamerican Institute for Cooperation on Agriculture, forerunner of CATIE, was established in 1942, and the land was established on what had long been a sugar cane farm. Similarly, in the absence of reliable data, an assumption that there is no significant soil C sequestration associated with tree growth is consistent with IPCC default accounting guidelines for land use change from grassland to trees (IPCC 2006).

Data were entered into the dairy and beef farm LCA model based on Styles et al. (2015) and adapted for Costa Rica by Mazzetto et al. (2020). That model applies an IPCC (2006, 2019) Tier 2 approach to calculate forage intake and consequent enteric methane (CH_4_) and manure management emissions of CH_4_ and N_2_O. Energy requirements are calculated for each animal cohort based on IPCC Tier 2 equations, and satisfied first by energy available in concentrate and other supplementary feeds. Residual energy requirements are then satisfied by grazed forages—specifically *Cynodon* and *Brachiaria* grasses with dry matter digestibilities of 60%. Gross energy and total N intake are then calculated based on known feed characteristics (INRA, CIRAD,, and FAO 2019) and used to calculate the next steps of the Tier 2 emissions equations. The IPCC (2006) method for enteric CH_4_ was modified with region-specific CH_4_ conversion factors (Y_m_) of 0.074, 0.073 and 0.057 for dairy cows, heifers and bulls, respectively (Mazzetto et al. 2020) (Table 2). These values are applied to the gross energy intake of the cattle to determine the fraction of energy lost as CH_4_, subsequently converted into a mass.

Ammonia (NH_3_) emissions and nitrate (NO_3_^-^) losses to water are based on best available emission factors for temperate dairy systems (Duffy et al. 2018; Misselbrook et al. 2015), reflecting a lack of emission factor development for tropical systems (Table 2). Nitrous oxide emissions from soils are calculated using an IPCC (2019) Tier 1 emission factor, based on N excreted on to pastures directly by grazing animals (Tier 2 approach), and fertiliser and manure N applications (Mazzetto et al. 2020). Indirect N_2_O emissions were calculated based on IPCC (2019) Tier 1 emission factors applied to volatilised NH_3_-N and leached N. Nitrogen excretion into housing, subsequently flowing into manure storage and field application (minus volatilisation losses) was calculated by cohort, based on lactating cows spending 6 h per day indoors, young stock being housed for 24 h, and dry cows, heifers and bulls being outdoors all of the time. The Tier 2 approach for N excretion (IPCC 2006) employs a mass balance and represents the difference between N intake calculated from feed and N incorporated into the tissue of growing animals and/or converted into milk protein. Finally, average C sequestration of 1.43 Mg C ha^−1^ year^−1^ measured in tree growth was extrapolated to the 62.1 ha of farm area, and converted into an annual CO_2_ offset at the farm level based on the molecular mass of CO_2_ (44) to C (12). This 325,298 kg CO_2_ year^−1^ offset (Table 2) was included as a negative emission in the land use change section of the farm LCA tool, and subsequently translated into an offset per kg FPCM produced on the farm through the same allocation procedures applied to other farm-level emissions.

To reflect uncertainty around emission factors and biomass C sequestration, error propagation was performed to estimate 95% confidence intervals. The square root of the sum of squared uncertainty ranges for major footprint components was calculated to derive a 95% confidence interval. Components were separated into high (±50%) and medium (±25%) levels of uncertainty, with bought animals, soil N_2_O, imported feed and biomass C sequestration categorised as high uncertainty, and enteric CH_4_, manure management and agrochemical and energy emissions categorised as medium uncertainty.

## Results and discussion

### GHG emissions from the various sources on and off the farm

**Fig. 4:**
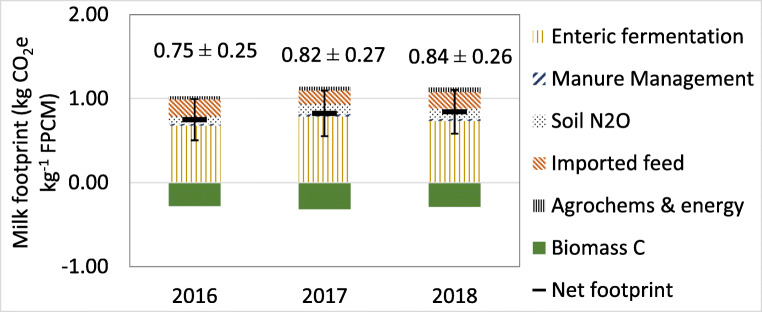
Breakdown of milk carbon footprint for each year, indicating gross and net (after biomass carbon offset) emissions, and 95% confidence intervals.

Annual farm system GHG emissions averaged 1197 Mg CO_2_ eq. year^−1^ between 2016 and 2018, dominated by enteric CH_4_ which averaged 794 Mg CO_2_ eq. year^−1^ (66%) over the 3 years (Table 2). Imported feed and soil emissions were the next major sources of GHG emissions, followed by agrochemical manufacture (most notably fertilisers) plus energy use (electricity, petrol and diesel), and manure management (Table 2, Fig. 4). Owing to the relatively small proportion of time during which animals are housed and the treatment of slurry from lactating cows in an anaerobic digester, manure management emissions (CH_4_ and N_2_O) were small, whilst N_2_O emissions from managed soils made a contribution of 10% to GHG emissions. Upstream life cycle emissions such as the production of concentrate feed and agrochemicals largely arise outside of Costa Rica, and whilst they represent an important contribution to the LCA-based product (milk) C footprint, they are not counted in Costa Rica’s national GHG inventory as per UN Framework Convention on Climate Change rules (UNFCCC 2016). Consequently, ‘inventoried’ emissions (i.e. emissions directly contributing to Costa Rica’s national annual reported emissions) from the farm system averaged 958 Mg CO_2_ eq. from 2016 to 2018, 20% less than the broader total system emissions. Net biomass sequestration in live fences over the 3-year study period offset 26–28% of total system life cycle emissions, and 33–35% of inventoried emissions (Table 2). Considering uncertainty ranges, biomass sequestration offset 21–37% of life cycle emissions related to 1 kg FPCM (Fig. 4).

Energy-based allocation results in approximately 84% of farm system emissions (and carbon offsets) being allocated to milk production, the remainder being allocated to animal live weight, representing the gross energy contained in the main products leaving the farm (i.e. milk and sold animal live weight). When divided by total milk outputs (Table 2), this resulted in an average gross milk footprint of 1.10 kg CO_2_ eq. per kg FPCM over the 2016 to 2018 study period, before offsets due to tree growth. These footprints are already comparatively small by international and Costa Rican standards (Soteriades et al. 2018; Mazzetto et al. 2020; Duffy et al. 2021) using the same LCA methods, reflecting the efforts at CATIE that have gone into livestock genetic improvement, manure management (anaerobic digestion) and grassland management, and the high potential for efficient production in tropical systems owing to year-round grass productivity and feed supplementation. After accounting for biomass C sequestration offsets, milk footprints were reduced to between 0.75 and 0.84 kg CO_2_ eq. per kg FPCM (Fig. 4).

The Tier 2 GHG accounting methods employed in the farm LCA tool are widely used, but are subject to some uncertainty, most notably with respect to feed intake. All animal and manure emissions are directly related to feed intake, which is calculated based on two somewhat uncertain parameters: (i) animal energy requirements, estimated from animal weight and productivity (live weight gain or milk yield) via IPCC (2006) equations; (ii) forage digestibility, which is highly variable across pastures based on, inter alia, species mix, climate and management factors. Whilst we apply the most relevant, grass species-specific digestibility factor and Latin American-specific *Ym* (fraction of energy intake lost as enteric methane) factors (IPCC 2006), there is some inherent uncertainty in footprint calculations that could influence the inferred offset potential of live fences, reflected in the 21–37% of gross footprint offset calculated from error ranges presented in Fig. 4.

### Contribution by trees

In September 2016 when the trees were first surveyed, there were 182 trees within the sampled plots. Based on the presence of foliage in 2016 (even if just sprouting) and subsequent condition in 2019, it was assessed that 104 of these were viable and contributed to CO_2_ sequestration over the 30-month interval (Supplementary Information). One plot (type s1) with six trees was subsequently removed by the farm due to an impending change in land use, and this plot was excluded from calculations.

In 2016, mean DBH for living *Erythrina fusca*, *Gliricia sepium* and *Tricanthera gigantea* were (standard error of mean in parenthesis) 13.5 (1.06) cm, 16.6 (1.96) cm and 10.8 (1.02) cm, respectively. In 2019, the respective values were 20.3 (1.65) cm, 19.8 (1.99) cm and 16.7 (2.80) cm. Mortality of *E. poeppigiana* was high and there were too few surviving specimens to make calculation of a mean meaningful. The C stock in living trees in 2016 was 14.8 Mg C ha^−1^ plus 0.78 Mg C in tree stumps without canopies (assumed to be dead). In 2019, C stock in trees was 18.4 Mg C ha^−1^ plus 0.81 Mg C in dead stumps. Net C sequestration rate was 1.43 Mg C ha^−1^ year^−1^ which required an uptake of 5.24 Mg CO_2_ ha^−1^ year^−1^ (net, after respiration). This rate of C sequestration is within the range of values presented by Nair et al. (2010), albeit for different species and systems from these reported here. Over 30 months, the mean increase in biomass for the most actively growing trees was 294% and 201% for *E. fusca* and for *G. sepium*, respectively, whereas mean overall biomass accumulation was only 24% due to tree mortality and slow recovery from pollarding. This illustrates the gap between potential and actual growth. Tree crown cover in live fences over the whole dairy farm was 30.0% (vertical projection), determined in 2016.

Survival of planted woody cuttings was low, particularly those in live fence type s0 planted in 2015 and 2016, because none had foliage at the time of first recording in 2016, and either they were cuttings which had failed or they did not survive recent pollarding (Table 1). By March 2019, only 104 trees were actively growing. Compared to trees recorded 30 months earlier, by 2019, 48% *E. fusca*, 75% *E. poeppigiana*, 18% *G. sepium* and 35% *T. gigantea* were dead, many of which had been recorded as having no foliage in 2016. *Erythrina fusca* is not commonly planted as a live fence in Costa Rica but is used on this farm because the level terrain leads to regular flooding of paddocks during the wet season, and *E. fusca* is known to be waterlogging tolerant. It also strikes readily from woody cuttings, so the reasons for the high mortality are not known. Mortality greatly affected biomass accumulation potential and emphasised the need to replace all dead cuttings which were supporting fencing wire, many of which had decomposed or been eaten by insects. These findings are in contrast to those of Zahawi (2005), where 2.0-m-long *E. fusca* cuttings were initially slow to establish but by 5 months, 85% had survived.

Taking into consideration the lost potential due to mortality, the low density of trees along live fences with simple structures (s0 and s1 in Table 1) and the 32% of the total length of fences which were post and wire which could be replaced by live fences, the potential for increasing C sequestration in actively growing trees is evident. On the dairy farm, there are 533 m fences (all types) ha^−1^ of land with an average of 5.0 m between each living tree in 2019. Reducing distance between trees to 2.5 m would double the tree density from 72 trees ha^−1^ in 2019 to 144 ha^−1^.

Over the period 2016–2018, mean milk production per cow was 18.1 kg FPCM year^−1^ (Table 2) and changed little despite woody biomass increasing by 24% over this period. This compares with a mean milk production per cow in specialist dairy farms in Costa Rica of 13.0 kg FPCM year^−1^ (Mazzetto et al. 2020). Although tree canopy cover was 30.0% in 2016, at CATIE, higher than the national average milk yields were achieved with a lower C footprint (Mazzetto et al. 2020; Duffy et al. 2021).

In a 3-year silvopastoral experiment in Brazil (Paciullo et al. 2014) which included 5- to 8-year-old *G. sepium* at 70 trees ha^−1^, in the first year, milk yields were higher in silvopastures compared to no-tree controls, and thereafter, there were no significant differences. At the CATIE dairy farm, doubling the population of trees is achievable and would not necessarily double the canopy land cover. Canopies of adjacent trees overlap and some fence lines are double rows, particularly fence types c1 and c2. The 32% of fences which are preserved dead posts and wire are being replaced by live fences as time and resources permit. It is reasonable to assume that a doubling of tree population would have little or no negative effect upon milk production, but due to increased CO_2_ sequestration, the footprint for milk would decrease yet further. At some point, there will be a trade-off between tree canopy cover and pasture production, but in the system studied here, that point did not seem to have been reached.

The unique aspect of this article is the integration of measurement of C sequestration in live fences over a 2.5-year interval in a tropical silvopastoral system into the C footprint of milk produced from that system. As there were only two measurement events, it is not possible to determine where on their growth curves the trees were, but as the majority were planted since 2010 they would be in their phase of rapid growth. Over 30 months, healthy *Erythrina fusca* trees almost trebled in biomass and *Gliricidia sepium* doubled. Nevertheless, at some point, the trees will approach maturity and growth rate will decrease.

Trees which died between the two recording events decomposed where they stood. These would return much of the C sequestered during tree growth back into the atmosphere, potentially leaving a mitigation deficit for growth of the next generation of trees to fill. In order to maintain the mitigation effect of sequestered C, the wood from any harvested trees removed would need to be planted as cuttings or incorporated into articles with a long lifespan, and/or used to substitute emission-intensive products and fuels, and/or undergo (future, end-of-life) bioenergy use coupled with carbon capture and storage (Forster et al. 2021). Such uses of wood are integral to projections for meeting net zero C targets (IPCC 2019). However, there is likely to be a trade-off between fast-growing tree species suited to live fences, such as *Erythrina* spp., and the production of high-quality timber needed for buildings. For example, *G. sepium* is suitable for structural construction, furniture and farm tools.

Trees provide numerous benefits besides C sequestration. It was evident from observing the behaviour of the cattle that during hot periods of the day, they sheltered under the canopies of the more mature live fences (Fig. 1). Cattle were observed browsing foliage from lower hanging branches. The benefits of these two services were not quantified during this study, but it is reasonable to assume that they benefitted the productivity of the farm.

### Reconciling the balance between C sequestration rates in live fences and the GHG emissions from dairy production

It should be noted that the empirical data collected during this study were tree stem diameters 30 months apart with inputs and outputs from the dairy farming system over 3 years, and that the conclusions were drawn from models using these data. Thus, the balance between C sequestration in live fences and GHG emissions from dairy production depends on the specific tree biomass accumulation and LCA models utilised. Models evolve as understanding develops. Those used in this paper, developed according to current standard protocols (ISO 14040 2006; IPCC 2006, 2019) and derived from empirical data for tree biomass accumulation (Kuyah et al. 2012a, 2012b) were applicable at the time of writing but it is possible that they may vary at some point in the future in the light of new findings.

The CATIE dairy farm studied here is a highly productive dairy system by international standards. On an area basis, milk output was 13–15 Mg FPCM ha^−1^ year^−1^ and system GHG emissions were 19–20 Mg ha^−1^ year^−1^. This compares with milk output of just over 8 Mg ha^−1^ year^−1^ and a GHG emission per unit of land of 12.5 Mg ha^−1^ year^−1^ for a typical grazing-based dairy farm in the UK (Soteriades et al. 2018). In terms of C footprints per kg FPCM, the CATIE farm is below the average of 1.4 kg CO_2_ eq. kg^−1^ FPCM calculated across specialist dairy farms in Costa Rica (Mazzetto et al. 2020; Duffy et al. 2021). For lower intensity, more extensive systems, the proportion of dairy system GHG emissions offset by live fences could be considerably higher. However, less intensive systems require more land to produce the same quantities of output, and there is a perverse risk that more extensive systems which can more easily be considered C neutral at the system level through C sequestration in soils and/or biomass may drive leakage of emissions through food production and agricultural expansion elsewhere, as has been illustrated previously (Plassmann 2011; Styles et al. 2015). Future evaluations need to look at more than just one indicator, such as both emissions and land use per unit of product over the same period.to take into account other benefits as well as trade-offs.

The CATIE dairy farm provides a useful template for sustainable intensification of dairy production in the tropics which combines efficient use of land to support land sparing (Lamb et al. 2016) with an important direct GHG offset through live fences. Such an approach could deliver habitat and biodiversity provisioning services at the landscape scale, thus also contributing aspects of land sharing as potentially being land sparing through increased production by area of land. Although pastures dominated by nutritionally poorer C4 grass species result in lower milk yields per cow compared to temperate systems utilising C3 ryegrass and clover mixtures, being located in a humid tropical zone confers many advantages such as grazing all year round and rapid tree growth. Caution should be exercised in extrapolating the findings here to highly seasonal locations such as the dry tropics (but which tend to be beef rather than dairy systems) and temperate climates, where trees cease to grow in the winter or dry season and housing or kraaling of cattle will be necessary at certain times of the year.

## Conclusions

In this paper, we show for the first time that with a moderate tree population, between one-quarter and one-third of the calculated GHG emissions (depending on the LCA models and inventory system employed) was offset by trees; on this farm in particular, there is clear scope for increasing tree population and C sequestration by replacing post and wire fences and those trees which failed to thrive. The implications are significant. The CATIE dairy farm is at the highly productive and intensive end of the scale in the tropics—in less intensive systems, a similar tree cover would deliver considerably greater proportional emissions offset with lower emissions per hectare of land. The farm exhibits the advantages accruing from both land sparing (high productivity per unit area of land) and land sharing (tree cover providing ecosystems services such as habitat provision and biodiversity) and may serve as a template for more environmentally benign livestock farming, at least in the humid tropics. However, the relatively high concentrate supplementation on the CATIE farm contributes to food-feed competition; thus, production systems that supplement less concentrate might be more sustainable in a circular food system and over the long term. The results also show that species selection, method of establishment and subsequent management have a significant effect upon the growth potential of trees and consequent ecosystem services provided, and these factors should be borne in mind when developing SPS. These will vary according to location, local climate, soil type, farming system and farmer aspirations, so accordingly, location-specific SPS need to be developed. Harvesting wood to supply cascading, bio-based value chains may provide an opportunity to extend the duration of active offsetting achieved by the wood produced, but this requires further research and development.

## Supplementary information

ESM 1 (XLSX 43 kb)

ESM 2 (XLSX 33 kb)

## Data Availability

All other data generated or analysed during this study are included in this published article (and its supplementary information files).
